# Processes linking cultural ingroup bonds and mental health: the roles of social connection and emotion regulation

**DOI:** 10.3389/fpsyg.2013.00052

**Published:** 2013-02-28

**Authors:** Nicole A. Roberts, Mary H. Burleson

**Affiliations:** School of Social and Behavioral Sciences, Arizona State UniversityGlendale, AZ, USA

**Keywords:** culture, ethnic identity, ingroup affiliation, emotion regulation, social connection, loneliness, Mexican American

## Abstract

Cultural and ethnic identities influence the relationships individuals seek out and how they feel and behave in these relationships, which can strongly affect mental and physical health through their impacts on emotions, physiology, and behavior. We proposed and tested a model in which ethnocultural identifications and ingroup affiliations were hypothesized explicitly to enhance social connectedness, which would in turn promote expectancy for effective regulation of negative emotions and reduce self-reported symptoms of depression and anxiety. Our sample comprised women aged 18–30 currently attending college in the Southwestern US, who self-identified as Hispanic of Mexican descent (MAs; *n* = 82) or as non-Hispanic White/European American (EAs; *n* = 234) and who completed an online survey. In the full sample and in each subgroup, stronger ethnocultural group identity and greater comfort with mainstream American culture were associated with higher social connectedness, which in turn was associated with expectancy for more effective regulation of negative emotions, fewer depressive symptoms, and less anxiety. Unexpectedly, preference for ingroup affiliation predicted *lower* social connectedness in both groups. In addition to indirect effects through social connection, direct paths from mainstream comfort and preference for ingroup affiliation to emotion regulation expectancy were found for EAs. Models of our data underscore that social connection is a central mechanism through which ethnocultural identities—including with one's own group *and* the mainstream cultural group—relate to mental health, and that emotion regulation may be a key aspect of this linkage. We use the term *ethnocultural social connection* to make explicit a process that, we believe, has been implied in the ethnic identity literature for many years, and that may have consequential implications for mental health and conceptualizations of processes underlying mental disorders.

## Introduction

Culture shapes us in many, if not most, ways. Through intergenerational transmission of attitudes, values, and beliefs (Matsumoto, 1993), culture influences how we relate to others, manage our emotions, and experience and express psychological distress. In addition to these relatively direct and specific cultural influences, the psychological relationships an individual has with his or her culture can have a considerable impact on mental health. For example, the degree of “fit” between a person and his or her culture's norms (e.g., norms of emotional expression) can play a role in mental health (Tsai et al., 2006; Chentsova-Dutton et al., 2010; Soto et al., 2012) as can the degree to which an individual embraces his or her cultural identity or identities (Phinney and Kohatsu, 1997; Lusk et al., 2010). Further, ethnic and cultural identities can have considerable influence over the relationships individuals seek out and how they feel and behave in these relationships. These connections in turn influence health directly and indirectly through the support they provide and through their impact on emotions, physiology, and behavior (for reviews see Kawachi and Berkman, 2001; Kiecolt-Glaser and Newton, 2001; Mikulincer and Shaver, 2007; Hawkley and Cacioppo, 2010; Roberts, 2012). The present paper discusses how identification, preference for, and comfort with members of one's own and other ethnocultural groups may shape emotional processes. We posit that ethnic and cultural ingroup contexts enhance one's sense of social and emotional connection, in turn facilitating emotion regulation and consequently better mental health.

In this paper, we adopt Matsumoto's (1993) broad definition of culture as, “A shared system of beliefs, attitudes, values, and behaviors communicated from one generation to the next via language” (p. 120), and ethnicity as self-reported membership in a group of origin. Although ethnic and cultural background may map onto one another, the two certainly are not isomorphic (Matsumoto, 1993). Individuals differ both in the extent to which they adopt the values of their culture(s) of origin (Tsai et al., 2006), and in the strength of their *ethnic identity*. As Phinney and Ong (2007) describe, “ethnic identity has been studied largely with reference to one's sense of belonging to an ethnic group, that is, a group defined by one's cultural heritage, including values, traditions, and often language” (Phinney and Ong, 2007, p. 274). Therefore, ethnic identity reflects an assessment of feelings about one's cultural identity, as well as racial/ethnic identity (Phinney, 1996). Although ethnic identity may vary by group insofar as it interacts with group-specific processes and values (Cokley, 2007) its foundation or core concept is largely pan-group or pan-cultural (Phinney and Ong, 2007).

Ethnic identity is a multifaceted, developmental construct that begins with self-identification as a member of a racial/ethnic group and expands to include investment in, commitment and attachment to, and/or pride in one's ethnic group of origin (see Phinney and Ong, 2007). Ample theory and data indicate that stronger ethnic group identity is associated with indicators of better mental health. In multiple ethnic groups (primarily in the US and Canada) and across individuals varying in immigration and generational status and age or cohort (adolescents, college students, community adults, and older adults), stronger ethnic identity is associated with less depression, anxiety, interpersonal daily hassles, and loneliness, and with more self-esteem, coping ability, mastery, optimism, and resiliency (Kim, 1999; Roberts et al., 1999; Tsai et al., 2001; Gaudet et al., 2005; Juang et al., 2006; Schwartz et al., 2007; Torres and Ong, 2010; Williams et al., 2012). A low level of ethnic identity even has been suggested as a potential risk factor for serious mental illnesses such as schizophrenia (Veling et al., 2010). Although ethnicity and ethnic identity typically are most salient for ethnic minority group members, psychological benefits of ethnic identity have been observed among majority (non-Hispanic White/European American) individuals as well (Phinney and Alipuria, 1990; Roberts et al., 1999).

Strong ethnic identity often is accompanied by a preference for socializing with members of one's own ethnocultural group (Malcarne et al., 2006); we refer to this latter phenomenon as *ingroup affiliation.* Both ethnic identity and ingroup affiliation appear to promote psychological health and well-being. Often-cited potential mechanisms for these effects include reducing acculturative stress (Gaudet et al., 2005; Schwartz et al., 2007), enhancing self-esteem (Tatum, 1997; Phelps et al., 2001; Mossakowski, 2003; Torres and Ong, 2010; Williams et al., 2012), providing social support (Noh et al., 1999; Noh and Kaspar, 2003), reducing loneliness (Kim, 1999; Roberts et al., 1999), and enhancing a sense of community embeddedness (Galliher et al., 2011; Kenyon and Carter, 2011; Rivas-Drake, 2012). For example, with respect to the acculturation process, maintaining stronger ethnocultural group affiliations can ease the adjustment to learning a new language, customs, and behaviors, and/or minimize the need to incorporate these new cultural aspects into one's daily life (e.g., Gaudet et al., 2005). More positive and supportive relationships within one's ethnocultural group also can encourage active coping approaches and in turn help mitigate the intensity of negative emotional reactions to racial discrimination (Noh et al., 1999; Noh and Kaspar, 2003) or anxiety regarding anticipated future discrimination (Soto et al., 2011). Finally, cultural pride in or regard for one's ethnic group can be associated with a sense of belonging or connection to others, enhanced self-esteem, reduced depression, and greater resiliency (Resnick et al., 1997; Jones and Galliher, 2007; Brown, 2008; Rivas-Drake, 2012).

On the other hand, several studies have not found relationships between ethnic identity or ingroup affiliation and positive mental health indicators, or have found inverse relationships (see Juang et al., 2006). These inconsistencies likely reflect cultural, historical, and contextual complexities. Relationships between ethnic identity and mental health are complicated by the fact that the multiple cultural identities participants may hold and the multiple contexts in which these identities play out may shape mental health (Juang et al., 2006). An ethnic minority individual with a strong ethnic identity may struggle in an environment that is not accepting of diverse behaviors and attitudes, particularly if the individual is from a marginalized and/or socially devalued group. For Latino students at primarily White universities, for example, there is an inverse relationship between ethnic identity and some indicators of psychological functioning (e.g., academic persistence; Castillo et al., 2006). A strong ethnic identity, therefore, may not uniformly protect mental health if the identity is stigmatized or promotes behavior that is incongruent with mainstream cultural values.

Further, separate mediating processes may operate in different directions, depending on the aspects of ethnic identity or cultural affiliations that are measured [e.g., relationship to one's culture of origin vs. mainstream culture as discussed in Birman and Taylor-Ritzler (2007)]. For example, models of acculturation suggest that the ability to achieve an adaptive bicultural identity, or to navigate between one's culture of origin and mainstream culture, reflects psychological flexibility (LaFromboise et al., 1993). This more “secure or mature” ethnic identity (Phinney et al., 2007, p. 480) then predicts better adaptation (Phinney et al., 2001). Among Mexican Americans, stronger identification with *both* Mexican and American cultures is associated with reduced feelings of alienation and loneliness (Suarez et al., 1997). Greater cultural flexibility also may be associated with more positive intergroup attitudes, which contribute to greater openness, awareness, and interpersonal connection (LaFromboise et al., 1993; Phinney et al., 2007). A balance therefore may be needed in terms of maintaining close affiliations with one's own ethnocultural group, but also developing comfort with other groups. For ethnic minority groups, this may mean greater comfort with the dominant or mainstream culture, and for majority groups, this may mean openness to other cultures (Phinney et al., 2007). Nevertheless, for ethnic minority group members in particular, closer ties to one's own group may be a key part of promoting mental health.

In considering the ethnic identity and mental health literature, it became apparent to us that *social connection* is a unifying construct implicit in many of these studies. Social connection is a subjective sense of feeling emotionally together with others (Hawkley et al., 2007). As with other homeostatic states (e.g., maintaining adequate body temperature, maintaining an absence of pain, hunger, or thirst; L. C. Hawkley, Pers. Communication, December 25, 2012), the contented state that is associated with social connection is perhaps most noticed when it is absent, namely in the form of distress from perceived social isolation or loneliness (Peplau and Perlman, 1982). In both cross-sectional and longitudinal studies, loneliness predicts—and therefore social connection may protect against—a host of negative mental and physical health outcomes, including depression, cardiovascular disease, and even earlier death (see Hawkley and Cacioppo, 2010). Accordingly, loneliness often is included as an indicator of poor adjustment or poor mental health in studies of ethnic identity or acculturation processes (Kim, 1999; Roberts et al., 1999; Birman and Taylor-Ritzler, 2007).

As mentioned above, several studies have identified social support as a potential mechanism through which ethnic identity may enhance mental health. We note that although social connection and social support overlap, they arguably are distinct constructs. While social support is multifaceted (see Barrera, 1986, for a review), its essence is largely the *perceived or actual* availability of family, friends, or another significant individual in the person's life, particularly during times of need (Zimet et al., 1988). Perceived social support—the aspect of social support described by Barrera (1986) as “the cognitive appraisal of being reliably connected to others” (p. 416) is similar to our view of social connection. Importantly, however, social connection is strongly related to health even after statistically controlling for social support, when the latter is defined in terms of availability of others when needed (Hawkley and Cacioppo, 2010). As Hawkley and Cacioppo describe, “perceived social isolation is tantamount to feeling unsafe, and this sets off implicit hypervigilance for (additional) social threat in the environment” (Hawkley and Cacioppo, 2010, p. 220). It would follow, therefore, that a greater sense of connection with one's own ethnocultural group, and a greater sense of psychological safety, would predict mental health and well-being on a number of levels. In other words, psychological benefits certainly may be derived from closer ties with one's ethnocultural group via actual or perceived social support in the face of stress, but an enhanced overall sense of social and emotional connection with others is perhaps even more important for mental health.

Social connectedness also appears to enhance *emotion regulation*, another process that is implicit in studies of ethnic identity and mental health. Emotion regulation can be defined broadly as “the processes by which individuals influence which emotions they have, when they have them, and how they experience and express these emotions” (Gross, 1998, p. 275). From a clinical perspective, regulating negative emotions is particularly important, namely being able to anticipate and manage feelings of emotional upset when they arise (Gratz and Roemer, 2004). Social connection is associated with better self-regulation in a number of arenas, including emotion. For example, socially connected participants demonstrate better executive control during tasks involving effortful attentional shifting (Cacioppo et al., 2000), and report more positive and less negative daily affect and social interactions (Hawkley et al., 2007). More effective emotion regulation also explains higher physical activity in socially connected, compared to lonely, mid-aged and older adults (Hawkley et al., 2009).

As proposed by Coan (2008), because humans are inherently social, the mere presence of other individuals reduces both the anticipated and actual cost of engaging with the environment, which in turn may assist with managing negative feelings. When others are present, therefore, additional resources become available for self- and emotion-regulation. This effect is augmented when there is greater connection or attachment, or a history of mutually-beneficial interaction (e.g., between friends or spouses; Coan, 2008; Sbarra and Hazan, 2008). We suggest here that perhaps the sense of social connection that comes from stronger identification, affiliation, and comfort with one's cultural groups may enhance the availability of resources for emotion regulation.

As discussed above, a strong ethnic identity and more frequent affiliations with ethnoculturally-similar others can facilitate coping, reduce acculturative stress, and offset negative psychological effects of discrimination or marginalization. We posit that these benefits are occurring by enhancing social connection and in turn resources for emotion regulation. In other words, stronger cultural group connections may create a psychological foundation from which emotion regulation becomes easier. Although to the best of our knowledge such a mechanism has not been made explicit, several lines of evidence indicate that ethnic identity and ingroup affiliation provide the type of shared understanding and sense of connection that may directly and indirectly affect emotion regulation.

First, it may be easier to decode the emotions of similar others. Elfenbein and Ambady (2003a,b) have described this as an “ingroup advantage” for emotional communication. This advantage is accounted for by cultural familiarity rather than racial or ethnic similarity *per se*, suggesting that emotional expression and understanding are shaped in subtle ways by cultural context (Elfenbein and Ambady, 2003a,b; Beaupré and Hess, 2006; Thibault et al., 2006; Elfenbein et al., 2007). In addition to greater ease of detecting outward emotional expression, greater emotional understanding among cultural ingroup members may occur as a result of shared meaning of emotions and the contexts in which they are evoked (Lutz, 1983; Markus and Kitayama, 1994, 2003; Mesquita and Leu, 2007; Shweder et al., 2008). In general, feeling understood can reduce negative emotion and possibly even facilitate emotional processing at a physiological level (Seehausen et al., 2012). In particular, racial or ethnic ingroup similarity may reduce anxiety or other negative emotions, as demonstrated both in laboratory research (Anderson, 1989; Vrana and Rollock, 1998; Soto et al., 2012) and therapy (Cabral and Smith, 2011; Chang and Yoon, 2011) settings. This benefit may arise due to actual or perceived similarity. As noted earlier, race, ethnicity, and culture are not synonymous; nevertheless, race may serve as a cue for shared experience (e.g., we are both Black and therefore share common ground; Tatum, 1997). Like actual similarity, perception of similarity may lead to greater openness and in turn greater social and emotional connection (Chang and Yoon, 2011). Ingroup similarity also can enhance positive emotion, such as through the use of ingroup humor (Pogrebin and Poole, 1988; Roberts and Levenson, 2006).

By definition, an “emotionally regulated” relationship is one that achieves a balance of more positive emotion and less negative emotion (Gottman and Levenson, 1992). Thus, we suggest that ethnic identity and ingroup affiliations may benefit emotion regulation directly by offering the type of comfort and shared understanding that can contribute to this positive–negative emotional balance, as well as indirectly by freeing cognitive and emotional resources to facilitate subsequent emotion regulation.

In sum, as discussed above, previous studies show that ethnic identification can increase one's sense of community, reduce loneliness, and ultimately contribute to mental health for both ethnic minority and majority individuals (e.g., Kim, 1999; Roberts et al., 1999; Galliher et al., 2011; Kenyon and Carter, 2011; Rivas-Drake, 2012). Therefore, we believe that social connection clearly is a central part of the benefits provided by ethnocultural identity and ingroup affiliations. As Phinney and Ong (2007) describe, the concept of ethnic identity reflects a sense of self plus a sense of belonging. One goal of the present study was to make more explicit the fact that social connection is an integral part of ethnocultural identity and affiliation, in order to examine more closely the “black box” (Birman and Taylor-Ritzler, 2007, p. 336) between relationships to one's culture(s) and mental health.

Notably, it is not only same-ethnic affiliations, but also cultural ingroup affiliations that may enhance social connection and promote better emotion regulation (e.g., Elfenbein and Ambady, 2003b). We expect, therefore, that among both ethnic majority and ethnic minority individuals, comfort with the mainstream or dominant culture also may be associated with these psychological benefits. Among ethnic minority individuals, a sense of integration into mainstream culture is associated with better psychological adjustment, as noted earlier (e.g., LaFromboise et al., 1993). Conversely, lack of comfort with mainstream culture or a sense that one does not belong may be associated with feelings of marginalization, alienation, and/or loneliness (e.g., Rivas-Drake, 2012). Among ethnic majority individuals, mainstream culture arguably *is* their primary culture (Helms, 1990), and therefore a greater sense of belonging to this environment should be associated with greater feelings of social connectedness and ease of emotion regulation. Even among non-Hispanic White individuals who identify with a European culture of origin (e.g., Irish, German; Martinez and Dukes, 1997), greater sense of belonging with mainstream culture still should be associated with stronger social connectedness and better emotion regulation.

Finally, the processes of social connection and emotion regulation closely map onto mental health outcomes (Schwartz and Olds, 1997; Berenbaum et al., 2003; Gratz and Roemer, 2004). Therefore, for the present study we were interested not only in examining the extent to which ethnic identity, preference for ingroup affiliations, and a sense of belonging to the larger cultural context (i.e., mainstream American culture) were associated with greater perceived social connection and better emotion regulation—but also how these in turn related to mental health indicators. In particular, we were interested in self-reported symptoms of depression and general feelings of anxiety, as these are widely reported in the ethnic identity literature and are reliable indicators of clinical disorders (mood and anxiety disorders and mental health problems more generally; Berwick et al., 1991).

### Overview of present study

Links between ethnic identity and mental health, and among social connection, emotion regulation, and mental health have been found in both minority and majority ethnic groups within the US. We note that ethnocultural group relationships may be a particularly powerful source of social connection, in turn shaping emotion regulation and mental health. Accordingly, the present study tested a model in which stronger ethnic identity, stronger preference for ingroup affiliation, and greater comfort with mainstream culture would be associated with (1) greater social connectedness (i.e., less loneliness), which in turn would predict (2) more effective negative emotion regulation. These in turn were anticipated to be associated with fewer self-reported depressive symptoms and lower self-reported levels of general feelings of anxiety. In other words, we predicted that the often-observed relationships between ethnic identity/ingroup affiliation and indicators of mental health would be mediated first by social connection and second by emotion regulation. We predicted that relationships between comfort with mainstream culture and indicators of mental health would be mediated similarly (although for slightly different theoretical reasons, as noted above).

We developed and refined our statistical model in a sample that included both non-Hispanic White/Caucasian/European American (*EA*) and Hispanic, specifically Mexican or Mexican American (*MA*) college women. We then tested the model separately in each of these two groups. With respect to comparing the model between the groups, on the one hand, many previous studies have found similar relationships between ethnic identity and outcomes such as adjustment, mental health, or loneliness, both in majority and among different minority groups. On the other hand, ethnic identity is more salient for ethnic minority group members, and processes such as collectivism, familism, and social connection may play a more prominent role for minority group members, such as Mexicans/Mexican Americans, than for majority group members in the US (Gaines et al., 1997). Therefore, although we expected more similarities than differences between the groups, we also expected some differences in the magnitude of the observed relationships. Specifically, based on previous research, we expected mean levels of ethnic identity and desire for ingroup affiliation to be higher, and mean levels of mainstream comfort to be lower, among MAs than EAs (e.g., Phinney and Alipuria, 1990; Roberts et al., 1999). We also expected stronger links between ethnic identity and social connection, and between preference for ingroup affiliation and social connection, in the MA group compared with the EA group.

We examined a Mexican/Mexican American (MA) group for several reasons. Hispanics/Latinos are the largest ethnic minority group in the US (17% of the US population), including in the Southwest where this study was conducted (30% of the state of Arizona population), and are one of the fastest-growing groups (second to Asians per 2010 US census data; US Census Bureau, 2012). Individuals of Mexican descent account for the majority of Hispanic/Latino population size and growth (US Census Bureau, 2011). Similar to other ethnic minority groups, Hispanics/Latinos report more psychological distress (e.g., feelings of sadness, hopelessness, worthlessness, feeling as though everything is an effort) than non-Hispanic Whites, and yet are less likely to receive mental health treatment (USDHHS, 2012). Also similar to the cultures of other ethnic minority individuals in the US, Mexican culture can be described as high on the values of collectivism and familism, meaning it values the welfare of one's larger community and particularly one's family (Gaines et al., 1997). Therefore, social connectedness is likely embedded in the value systems of this group, as well as other minority groups.

We studied college students, as they (along with adolescents) are the predominant focus for studies on ethnic identity. College students are experiencing a considerable transition, where ethnic identity and social-emotional connections with peers may be pivotal in shaping health and well-being (Phinney, 1992; Roberts et al., 1999; Phinney et al., 2007; Rivas-Drake, 2012). Given that the age of onset of many mental disorders (mid-teens to mid-20s; Kessler et al., 2007; de Girolamo et al., 2012) coincides with the transition to college, it may be particularly important to identify ways that individuals in this demographic can foster strong emotional connections with others and in turn bolster psychological resources to regulate their own emotions and behavior. Finally, we focused on women only, as they are more likely than men to seek social support at times of stress (Taylor et al., 2000), to be affected emotionally by distressed relationships (Gottman and Levenson, 1992), and to suffer from mood and anxiety disorders (Nolen-Hoeksema and Girgus, 1994; Lewinsohn et al., 1998; McLean et al., 2011).

## Method

### Participants

The entire sample comprised 316 women ranging in age from 18 to 30 years (*M* = 21.3, *SD* = 2.8). All participants were college students at a university in the Southwest and all had attended high school in the United States (US). A large majority of the sample (72.9%) were raised in the Southwestern US (1.3% were raised in Mexico). Education level varied less than would that of a community sample. All of the participants were college students; 68 (21.5%) reported having an Associate's degree, 14 (4.4%) reported having a Bachelor's degree, and 1 (0.3%) reported “some postgraduate college.” None reported attending a trade or business school. The median household income was $30,000–$49,999 per year, with 81.3% of participants reporting between $10,000 and $149,999 per year.

Within this larger group we studied two subsamples, representing an ethnocultural majority group and a relatively uniform ethnocultural minority group. As discussed below, we assessed race/ethnicity using wording adapted from the US Census. The majority group comprised 234 women who selected “Caucasian/White/European American” as their race and reported that they were not of Hispanic descent (*EAs*). The minority group comprised 82 women who identified themselves as “Hispanic/Latino of Mexican ancestry” (*MAs*). We assessed racial identification per census categories, but we note that particularly for Latinos, these categories may not make sense culturally or in terms of ethnic identification. The racial proportions of the MA group were as follows: 30 (36.6%) Caucasian/White/European American; 1 (1.2%) Asian American (who reported her race as “Asian and Latino”); 34 (41.5%) who selected “other” and wrote in Hispanic, Latino, or Mexican; 1 (1.2%) who selected “other” but did not provide additional information; and 16 (19.5%) missing.

### Procedure

Participants enrolled in an online survey administered through a secure website, “SurveyMonkey.com.” Participants received credit toward their course research requirements. The measures described here were administered along with measures collected for other studies. All procedures were approved by the university Institutional Review Board and were conducted in accordance with APA ethical guidelines.

### Measures

#### Demographics

Demographic measures included age, generational status, country of origin, country and, if US, region of upbringing, Hispanic/Latino descent (if yes, specific Hispanic/Latino background), racial category/identification, level of education, location of high school attendance, and annual household income.

#### Ethnic experience

To assess ethnocultural identity, preference for affiliation with one's own ethnocultural group, and comfort with mainstream US culture, we used the three corresponding subscales of the Scale of Ethnic Experience (SEE; Malcarne et al., 2006). The ethnic identity subscale comprises 12 items, such as “I have a strong sense of myself as a member of my ethnic group” and “My parents gave me a strong sense of cultural values.” The social affiliation subscale (which, for clarity of presentation, and based on the content of the items, we refer to as *ingroup affiliation preference*) comprises five items, such as “I think that friendships work best when people are from the same ethnic group” and “I find it easiest to trust people from my own ethnic group.” The mainstream comfort subscale comprises six items, such as “I do not feel a part of mainstream American culture” and “I understand how to get along well in mainstream America.” Responses range from 1 (*strongly disagree*) to 5 (*strongly agree*). Item ratings are reverse-scored where necessary and averaged to produce scale scores in which higher values represent higher levels of ethnic identity, preference for same-group affiliation, and comfort with mainstream culture.

The SEE was developed to assess these constructs regardless of the respondents' ethnicity, and was validated by the authors in a number of ethnic groups, including Mexican Americans and Caucasian Americans. In a previous study, 6-week test-retest reliabilities for the ethnic identity, ingroup affiliation preference, and mainstream comfort subscales were 0.86, 0.77, and 0.83 for Mexican Americans and 0.77, 0.59, and 0.77 for Caucasian Americans (Malcarne et al., 2006). In the current study, Cronbach's alpha coefficients for all three subscales were calculated in the full sample and within the two subsamples. All alpha coefficients were above 0.80.

#### Social connectedness

The revised UCLA Loneliness Scale (UCLA-R, Version 3) is a 20-item general measurement of loneliness that does not use the terms “lonely” or “loneliness” in any of its items, yet has high internal consistency, test-retest reliability, and concurrent validity (Russell, 1996). Abbreviated versions of the scale have been validated in Hispanic populations (Higbee and Roberts, 1994; Suarez et al., 1997; Alcorta et al., 2008). Responses refer to how often the respondent feels a particular way, and range from 1 (*never*) to 4 (*always*). Scale scores are strongly positively correlated with direct reports of loneliness, and strongly negatively correlated with direct measures of social interaction and close relationships. For example, loneliness is negatively related to other measures of social connectedness, including both perceived and actual social embeddedness (Russell et al., 1980), relationship satisfaction (Flora and Segrin, 2000), and responsiveness and other relationship-building social skills (Wittenberg and Reis, 1986). Furthermore, half of the items on the UCLA-R were written explicitly to assess satisfaction with social relationships (Russell et al., 1980), such that it has been referred to as measuring loneliness-connectedness (Hawkley et al., 2005), or social connectedness (Hawkley et al., 2012). Therefore, we reverse-scored the scale so that higher scores indicate greater social connectedness. Reliability was high in our full sample (α = 0.94), as well as in the EA (α = 0.94) and MA (α = 0.93) subsamples.

#### Emotion regulation

As our indicator of emotion regulation we used a shortened version of the Negative Mood Regulation (NMR) expectancy scale (Catanzaro and Mearns, 1990).^1^ This scale was designed to measure the respondent's self-efficacy in the use of general, behavioral, and cognitive strategies for regulating negative emotions and feelings. Respondents are asked how strongly they endorse statements about their beliefs regarding their ability to regulate negative affect under a number of conditions. For example: “When I'm upset, I believe that I can usually find a way to cheer myself up” or “When I'm upset, I believe that the advice friends give me won't help me feel better” (reverse-scored). Responses range from 1 (*strongly disagree*) to 5 (*strongly agree*). The scale has been used in multicultural samples (e.g., Gross and John, 2003) and a Spanish version was recently validated in Chile (Pfeiffer et al., 2012). We selected four indicators for each of the three subscales to produce a 12-item scale, which was scored such that higher values indicate greater expectations for successful regulation. Reliability for the overall score was high in our full sample (α = 0.86), as well as in the EA (α = 0.87) and MA (α = 0.86) subsamples. To correspond with current accepted definitions of “mood” and “emotion” (Beedie et al., 2005), we refer to this scale as measuring negative emotion regulation expectancy. Self-efficacy with respect to one's ability to regulate negative emotions is likely related to one's actual emotion regulation. For example, previous research shows that higher NMR scores are associated with reports of more frequent use of cognitive reappraisal (Gross and John, 2003), less frequent use of emotion suppression (Gross and John, 2003) and less avoidance of emotional experience (Brockmeyer et al., 2012). NMR scores also predict fewer symptoms of depression and anxiety (Kassel et al., 2007; see Pfeiffer et al., 2012, for a discussion of NMR expectancies across a number of clinical and non-clinical populations).

#### Depressive symptoms

To measure depressive symptoms, we used the Center for Epidemiologic Studies Depression scale (Radloff, 1977), a 20-item self-report scale that assesses the frequency of occurrence during the past week of both feelings and behaviors typical of depression. Responses on the CES-D range from 1 (*rarely; less than one day*) to 4 (*most of the time; 5–7 days*). We deleted the item that refers to loneliness in order to reduce spurious correlation between the UCLA-R and the CES-D, then averaged the values such that high scores represent more symptoms. A recent meta-analysis of factor structures of this scale among different ethnic groups suggests that the somatic and depressed affect scales differ between White and Latino subsamples (Kim et al., 2011), therefore we computed subscale values using the best item subsets for both of these groups (Kim et al., 2011) in order to determine whether relationships in the proposed models differed accordingly. Reliability for the 19-item score was high in our full sample (α = 0.91), as well as in the EA (α = 0.92) and MA (α = 0.89) subsamples.

#### Anxiety feelings

We used the six-item short form of the State Trait Anxiety Inventory (Marteau and Bekker, 1992) to assess general feelings of anxiety. This version has been used in Latino populations (e.g., Kachikis and Breitkopf, 2012) and the longer version has been validated in a Hispanic subpopulation (Novy et al., 1993). Respondents are asked to rate how strongly they *usually* feel a particular way; responses range from 1 (*not at all*) to 4 (*very strongly*). Reliability was high in our full sample (α = 0.86), as well as in the EA (α = 0.87) and MA (α = 0.84) subsamples.

### Data analysis

In order to simultaneously examine multiple hypothesized paths of direct and indirect influence among the variables, we tested path models in a structural equation framework using manifest variables. These analyses were carried out with Mplus 6.11 statistical software (Muthén and Muthén, 1998–2011), using maximum likelihood estimation. We report standardized path coefficients and their levels of significance, along with several global indices that, when used in conjunction with one another, can evaluate how well a model-implied covariance matrix fits the observed covariance matrix (Chen et al., 2008). The fit indices and their recommended values are described as follows: (1) a chi-square test for the difference in fit between the proposed model and a saturated model (with all possible paths among the variables). Small, non-significant values of the chi-square indicate good fit. (2) The root mean square error of approximation (RMSEA, Steiger and Lind, 1980). This test is an estimate of the discrepancy between the model covariance estimates and the observed covariances. Cutoffs are suggested such that values below 0.08 are acceptable, whereas values below 0.05 indicate good fit (Browne and Cudeck, 1993). (3) The comparative fit index (CFI; Bentler, 1990), which indicates the extent to which the model better fits the data than a “base” model constraining all of the variables to be uncorrelated with one another. Values greater than 0.95 indicate good fit (Hu and Bentler, 1999).

## Results

### Preliminary analyses

We compared the EA and MA subsamples on a number of demographic variables, including age, education, income, region of upbringing, and generational status, as well as on the other primary variables in the study. The groups did not differ in age (for EA, *M* = 21.3 years, *SD* = 2.8 years; for MA, *M* = 21.4 years, *SD* = 2.9 years) or education (median for both groups = “some college”). They did, however, differ in household income, linear χ^2^(1) = 7.02, *p* = 0.008. The median income ranges differed (EA = $50,000–$79,999; MA = $30,000–$49,999), and the MA group had disproportionately more members in the lower income ranges and disproportionately fewer members in the higher income ranges than the EA group. The two groups also differed in their region of upbringing, χ^2^(5) = 22.66, *p* < 0.001. Although a large majority of both groups were raised in the Southwestern US, proportionately more of the MA group fit this description. Finally, the two groups differed in their generational status, χ^2^(4) = 105.90, *p* < 0.001. As expected, the generational status of the MA group was lower, although both groups displayed some variability. For the EA group, 9 participants (3.8%) were born outside the US (8 in Eastern Europe, and 1 in Canada), 12 (5.2%) were born in the US with at least one parent born outside the US, and 212 (91.0%) were born in the US with both parents also born in the US [for 161 of this 212 (69.1% of the entire EA group), both sets of grandparents were also born in the US]. For the MA group, 8 (9.8%) were born in Mexico, 42 (51.2%) were born in the US with at least one parent born in Mexico, and 32 (39%) were born in the US with both parents also born in the US [for 16 of this 32 (19.5% of the entire MA group), both sets of grandparents were also born in the US].

Means and standard deviations of the ethnic experience, social connectedness, emotion regulation, depressive symptoms, and anxiety variables are shown in Table 1, for the overall sample and each of the two subsamples. Bivariate correlations among the study variables, separately for all participants and the MA and EA subgroups, are shown in Tables 2 and 3. The two-independent subsamples (MA vs. EA) were compared using analysis of variance, which revealed, as expected, that the MA group reported significantly higher ethnic identity than the EA group, *F*_(1, 314)_ = 24.70, *p* < 0.001, *partial* η^2^ = 0.070, and significantly lower mainstream comfort than the EA group, *F*_(1, 314)_ = 4.24, *p* = 0.040, and *partial* η^2^ = 0.013. In addition, ethnic identity and mainstream comfort were significantly negatively correlated in the MA group (*r* = −0.40, *p* < 0.001), but not related in the EA group (*r* = 0.06, ns). These findings are similar to those from other studies, suggesting that ethnic identity is more salient in minority than majority groups (Weisskirch, 2005; Brouillard and Hartlaub, 2006), and that mainstream comfort likely differs in meaning between the two groups. Contrary to expectation, the two groups did not differ significantly in preference for ingroup affiliation (*F* < 1.0). The groups also did not differ in levels of social connection, negative emotion regulation expectancy, depressive symptoms, or anxiety feelings (all *F*s < 2.5).

**Table 1:** **Means and standard deviations for study variables among all participants, non-Hispanic White/European American (EA) participants, and Mexican/Mexican American (MA) participants**.

	**All participants**		**EA participants**		**MA participants**	
	**Mean**	**SD**	**Mean**	**SD**	**Mean**	**SD**
Ethnic identity	3.3	0.8	3.2_a_	0.7	3.7_b_	0.8
Ingroup affiliation preference	2.3	0.9	2.3_a_	0.9	2.3_a_	0.8
Mainstream comfort	4.1	0.8	4.1_a_	0.8	3.9_b_	0.8
Social connection	3.1	0.6	3.1_a_	0.6	3.1_a_	0.6
Negative emotion regulation	3.8	0.7	3.7_a_	0.7	3.8_a_	0.7
Depressive symptoms	1.7	0.5	1.7_a_	0.5	1.6_a_	0.5
Anxiety feelings	2.2	0.7	2.2_a_	0.7	2.1_a_	0.6

*Note: All participants are female. For all participants, N = 289–316; for EA, N = 215–234; for MA, N = 74–82. Groups are not mutually exclusive except for EA vs. MA. For tests of group differences between EA and MA, means with different subscripts are significantly different, p < 0.05*.

**Table 2:** **Pearson or Spearman correlations among study variables for all participants**.

	**Age**	**Household income^a^**	**Generation in US^a^**	**Ethnic identity**	**Ingroup affiliation preference**	**Mainstream comfort**	**Social connection**	**Negative emotion regulation**	**Depressive symptoms**
Household income^a^	−0.17^**^	–							
Generation in US^a^	−0.04	0.17^**^	–						
Ethnic identity	−0.06	−0.08^*^	−0.22^***^	–					
Ingroup affiliation preference	−0.04	−0.09^†^	−0.15^**^	0.19^**^	–				
Mainstream comfort	0.04	0.09^*^	0.26^***^	−0.18^**^	−0.01	–			
Social connection	−0.08	0.07	0.09^*^	0.16^**^	−0.17^**^	0.14^*^	–		
Negative emotion regulation	0.06	−0.01	0.07	0.10	−0.23^***^	0.22^***^	0.60^***^	–	
Depressive symptoms	−0.01	−0.01	−0.11^*^	−0.13^*^	0.10^†^	−0.21^***^	−0.65^***^	−0.61^***^	–
Anxiety feelings	0.11^†^	−0.04	−0.10^*^	−0.13^*^	0.10^†^	−0.15^**^	−0.59^***^	−0.46^***^	0.60^***^

*Note: Ns range from 280 to 316. All participants female*.

a *Correlations with these variables are Spearman rho coefficients*.

† p < 0.10;

* p < 0.05;

** p < 0.01;

*** *p < 0.001*.

**Table 3:** **Pearson or Spearman correlations among study variables for non-Hispanic White/European American (Lower Triangle) and for Mexican/Mexican American (Upper Triangle) participants**.

	**1**	**2^a^**	**3^a^**	**4**	**5**	**6**	**7**	**8**	**9**	**10**
1. Age	–	−0.07	−0.06	0.17	0.05	0.16	0.16	0.13	−0.23^*^	−0.00
2. Household income^a^	−0.26^***^	–	0.20^†^	−0.09	−0.28^*^	0.18	0.08	0.03	0.02	−0.02
3. Generation in US^a^	−0.02	0.06	–	−0.18	−0.31^**^	0.35^**^	0.17	0.18	−0.16	−0.29^**^
4. Ethnic identity	−0.17^*^	−0.06	−0.10	–	0.39^***^	−0.40^***^	0.06	−0.04	−0.09	−0.01
5. Ingroup affiliation preference	0.07	−0.05	−0.19^**^	0.13^*^	–	−0.24^*^	−0.28^*^	−0.27^*^	0.09	0.20^†^
6. Mainstream comfort	0.01	0.08	0.27^***^	−0.06	0.06	–	0.23^*^	0.25^*^	−0.10	−0.18
7. Social connection	−0.17^*^	0.11	0.13^*^	0.20^**^	−0.13^*^	0.11^†^	–	0.70^***^	−0.68^***^	−0.59^***^
8. Negative emotion regulation	0.03	0.01	0.14^*^	0.13^†^	−0.22^**^	0.22^**^	0.56^***^	–	−0.63^***^	−0.59^***^
9. Depressive symptoms	0.06	−0.03	−0.21^**^	−0.13^†^	0.10	−0.25^***^	−0.64^***^	−0.60^***^	–	0.55^***^
10. Anxiety feelings	0.15^*^	−0.07	−0.19^**^	−0.15^*^	0.07	−0.15^*^	−0.59^***^	−0.41^***^	0.61^***^	–

*Note: Lower triangle comprises correlations among non-Hispanic White/European American (EA) women; Ns range from 209 to 234. Upper triangle comprises correlations among Mexican/Mexican American (MA) women; Ns range from 71 to 82*.

a *Correlations with these variables are Spearman rho coefficients*.

† p < 0.10;

* p < 0.05;

** p < 0.01;

*** *p < 0.001*.

### Focused analyses

We first developed a path model in all participants (*N* = 312), and then tested it in the two subsamples. The initial fully-mediated model was specified as follows: primary predictors in the model were ethnic identity, preference for ingroup affiliation, and mainstream comfort.^2^ Potential mediators were social connection and negative emotion regulation expectancy. Outcomes were depressive symptoms and anxiety feelings. The strongest form of our theoretical position suggests that social connection is an integral result of one's psychological relationship with one's culture, and that social connection is the pathway through which ethnic experience influences emotion regulation, which in turn influences mental health outcomes. Accordingly, structural paths were specified from the predictors to social connection, from social connection to negative emotion regulation expectancy, and from negative emotion regulation expectancy to the outcomes. Exogenous variables were allowed to correlate, as were outcomes.

**Figure 1:**
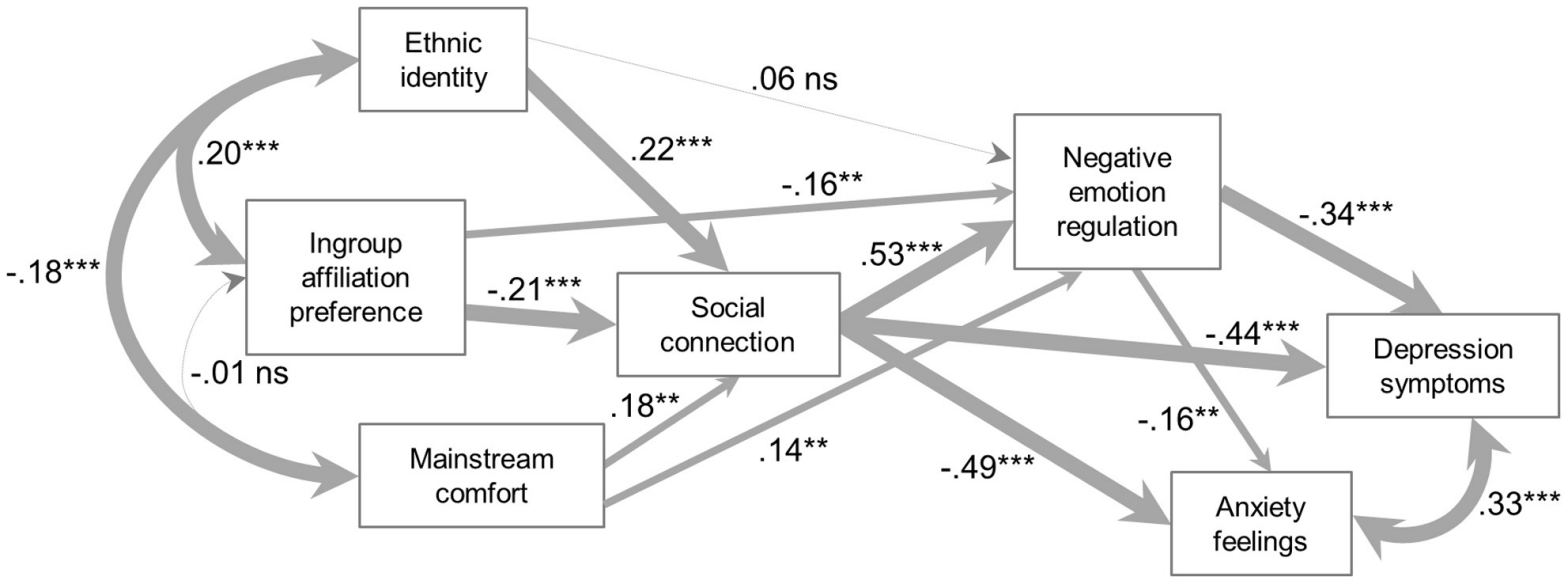
**All participants (*N* = 312)**.

The fit of this most stringent model (Model 1) to the data was unacceptable: χ^2^ = 121.177, *df* = 11, *p* < 0.000; RMSEA = 0.179, CFI = 0.795. We therefore first relaxed the requirement that negative emotion regulation expectancy fully mediate any relation between social connection and mental health outcomes by specifying direct structural paths from social connection to depressive symptoms and anxiety feelings. While definitely improved over Model 1, the fit of this model (Model 2) to the data was not ideal: χ^2^ = 25.795 1, *df* = 9, *p* = 0.0022; RMSEA = 0.077, CFI = 0.969. Therefore, we tested a third model (Model 3) in which we relaxed the requirement that social connection *fully* mediate any relationship between the initial predictors and negative emotion regulation expectancy. We made this decision based on the notion that one's ethnocultural identity/affiliation may influence emotion regulation or mental health through mechanisms such as enhancing self-esteem, which may bypass social connection. The fit of Model 3 to the data was excellent: χ^2^ = 7.899, *df* = 6, *p* = 0.2456; RMSEA = 0.032, CFI = 0.996. Standardized parameter estimates for this model are reported in Figure 1. Also, as can be seen in Table 4, Model 3 accounted for significant variance in both mediators and both outcomes.

**Table 4:** **Percentage of variance explained by Model 3, by group**.

	**Outcome**			
	**Depressive symptoms**	**Anxiety feelings**	**Social connection**	**Negative emotion regulation**
All participants (*N* = 312)	48.6^***^	36.2^***^	9.3^**^	38.7^***^
EA group (*N* = 234)	49.5^***^	36.1^***^	8.5^*^	37.4^***^
MA group (*N* = 82)	50.6^***^	38.7^***^	17.2^*^	46.4^***^

* p < 0.05;

** p < 0.01;

*** *p < 0.001*.

#### Model fit in ethnic majority and ethnic minority subsamples

**Figure 2:**
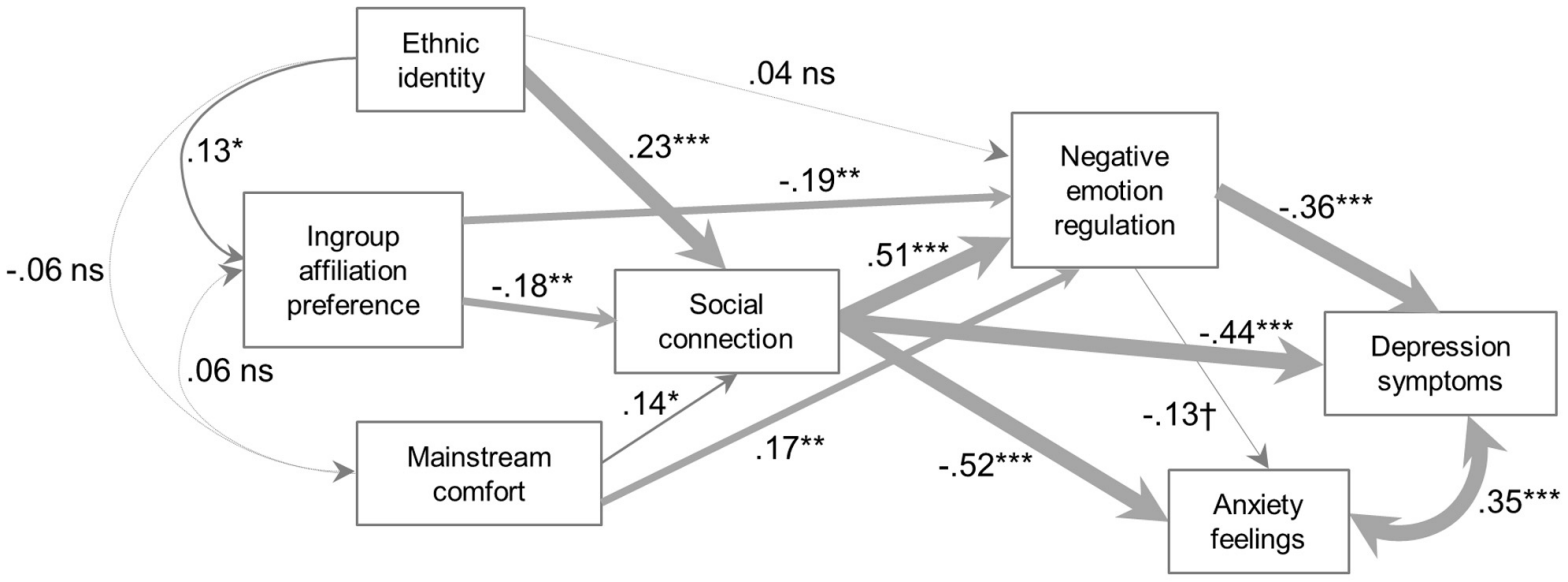
**Non-Hispanic White/European American (EA) participants (*N* = 234)**.

**Figure 3:**
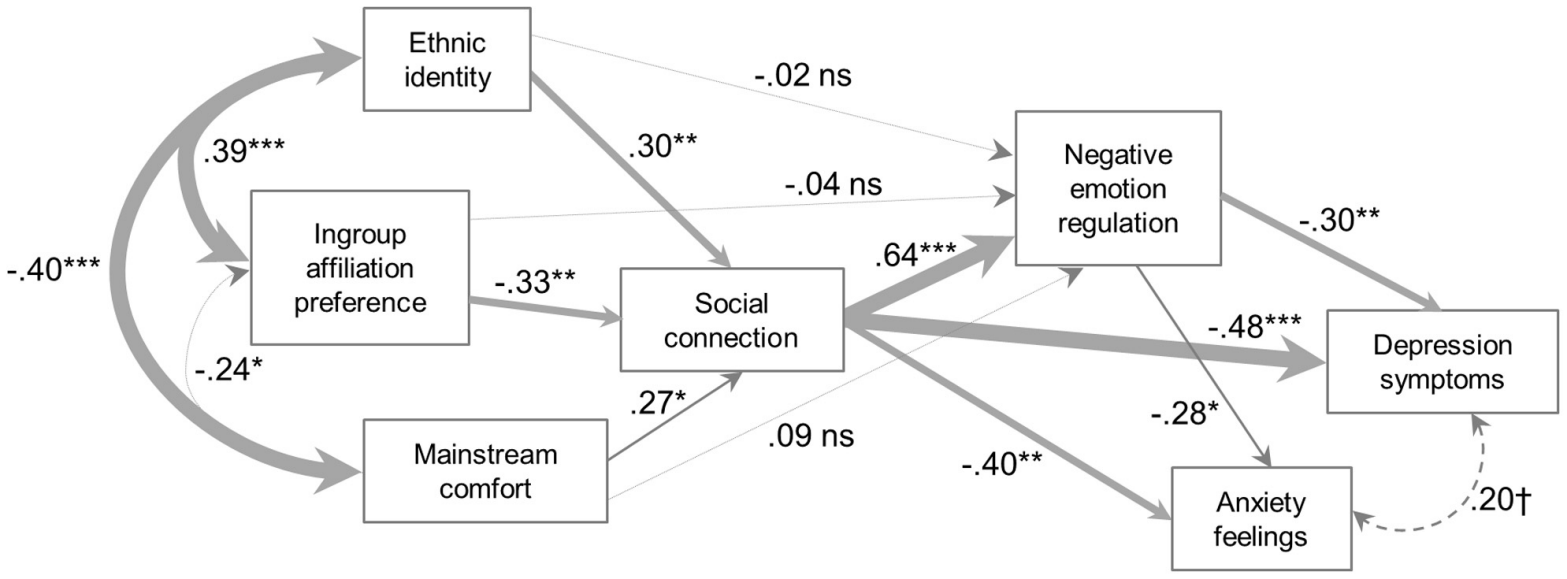
**Mexican/Mexican American (MA) participants (*N* = 82)**.

We next tested Model 3 in the two subgroups of our sample. As described earlier, the first subsample (EA) included women who self-identified as Caucasian/White/European-American, were raised in the US, and did not describe themselves to be of Latino or Hispanic descent (*N* = 234). This group was considered to be an ethnocultural majority subsample. Model 3 fit these data very well: χ^2^ = 9.146, *df* = 6, *p* = 0.1655; RMSEA = 0.047, CFI = 0.992; see Figure 2 for standardized parameter estimates. The second subsample (MA) comprised women who self-identified as Hispanic/Latino of Mexican descent (*N* = 82). For this subsample, the model fit also was excellent, χ^2^ = 3.337, *df* = 6, *p* = 0.7655; RMSEA = 0.000, CFI = 1.000; see Figure 3. Model 3 also explained significant variance in the mediators and outcomes in both subsamples (see Table 4).

As can be seen in the figures, there were differences in the path coefficient estimates between the two groups. Most notably, in the EA group, there were significant direct paths between ingroup affiliation preference and negative emotion regulation expectancy, and between mainstream comfort and negative emotion regulation expectancy. In the MA group, these paths were non-significant, suggesting that any influence of ingroup affiliation preference or mainstream comfort on negative emotion regulation expectancy occurred through an effect on social connectedness. It was also the case that the relation between ingroup affiliation preference and social connectedness was larger in the MA group, although we did not perform a statistical test on this difference. Another clear difference is seen in the relations among the ethnocultural experience variables: in the MA group, these variables were all significantly related, whereas in the EA group, only ethnic identity and ingroup affiliation preference were significantly related, and the relationship was small. A final difference is in the correlation between depressive symptoms and anxiety feelings, which was large and significant in the EA group, but small and only marginally significant in the MA group, suggesting that the meaning of depressive symptoms and general anxiety feelings may differ between the groups.^3^

#### Indirect effects

For comparison between groups, Model 3 indirect effects are reported for the EA and MA subsamples. Within each group, total indirect effects were significant or highly significant for each ethnocultural variable in predicting both depressive symptoms (Table 5) and anxiety feelings (Table 6). Overall, both total and specific indirect effects were stronger for depressive symptoms than anxiety feelings.

**Table 5:** **Standardized path coefficient estimates for model 3 indirect effects on depressive symptoms**.

	**Mediators**			
	**Social connection**	**Emotion regulation**	**2-level (both)**	**Total**
**PREDICTOR: ETHNIC IDENTITY**				
Full sample	−0.10^***^	−0.02	−0.04^**^	−0.16^***^
EA group	−0.10^**^	−0.01	−0.04^**^	−0.16^***^
MA group	−0.14^*^	−0.01	−0.06^†^	−0.19^*^
**PREDICTOR: INGROUP AFFILIATION PREFERENCE**				
Full sample	0.09^***^	0.05^**^	0.04^**^	0.18^***^
EA group	0.08^**^	0.07^**^	0.03^*^	0.18^***^
MA group	0.16^*^	0.01	0.06^*^	0.23^**^
**PREDICTOR: MAINSTREAM COMFORT**				
Full sample	−0.08^**^	−0.05^**^	−0.03^**^	−0.16^***^
EA group	−0.06^*^	−0.06^**^	−0.03^*^	−0.15^**^
MA group	−0.13^*^	−0.03	−0.05^†^	−0.21^**^

*Note: “Social connection” and “emotion regulation” refer to indirect paths with only the named variable as a mediator. “2-level (both)” refers to indirect paths with first social connection and then negative emotion regulation expectancy as sequential mediators. “Total” refers to the sum of all indirect paths. EA, non-Hispanic White/European American; MA, Mexican/Mexican American*.

† p < 0.10,

* p < 0.05,

** p < 0.01,

*** *p < 0.001*.

**Table 6:** **Standardized path coefficient estimates for model 3 indirect effects on anxiety feelings**.

	**Mediators**			
	**Social connection**	**Emotion regulation**	**2-level (both)**	**Total**
**PREDICTOR: ETHNIC IDENTITY**				
Full sample	−0.11^***^	−0.01	−0.02^*^	−0.14^***^
EA group	−0.12^**^	−0.01	−0.02	−0.14^***^
MA group	−0.12^*^	0.01	−0.05	−0.17^*^
**PREDICTOR: INGROUP AFFILIATION PREFERENCE**				
Full sample	0.10^***^	0.03^*^	0.02^*^	0.15^***^
EA group	0.09^**^	0.02	0.01	0.13^**^
MA group	0.13^*^	0.01	0.06^†^	0.20^**^
**PREDICTOR: MAINSTREAM COMFORT**				
Full sample	−0.09^**^	−0.02^*^	−0.02^*^	−0.13^***^
EA group	−0.07^*^	−0.02	−0.01	−0.10^**^
MA group	−0.11^*^	−0.02	−0.05	−0.18^*^

*Note: “Social connection” and “emotion regulation” refer to indirect paths with only the named variable as a mediator. “2-level (both)” refers to indirect paths with first social connection and then negative emotion regulation expectancy as sequential mediators. “Total” refers to the sum of all indirect paths. EA, non-Hispanic White/European American; MA, Mexican/Mexican American*.

† p < 0.10,

* p < 0.05,

** p < 0.01,

** *p < 0.001*.

In each ethnocultural group, specific indirect effects from ethnic identity and mainstream comfort through social connection to depression and anxiety were significant, such that stronger ethnic identity and mainstream comfort predicted less depression and anxiety. The more complex indirect effects including social connection as the first mediator and adding negative emotion regulation expectancy as a second mediator in sequence were necessarily not as strong (due to multiplication of fractional path coefficients); although paths to depression remained significant or marginally significant, paths to anxiety became non-significant. Also for both groups, when negative emotion regulation expectancy was the only mediator, paths from ethnic identity to both depression and anxiety were not significant, nor were paths from mainstream comfort to anxiety, although mainstream comfort remained a significant negative predictor of depressive symptoms in the EA, but not the MA, group.

Also in each of the EA and MA groups, specific indirect effects from ingroup affiliation preference through social connection to depression and anxiety were significant, such that stronger preference for ingroup affiliation was associated with more depression and anxiety. As with the other predictors, indirect effects including social connection as the first mediator and negative emotion regulation expectancy as the second mediator in sequence were necessarily not as strong, particularly for anxiety. With negative emotion regulation expectancy as the only mediator, indirect paths from ingroup affiliation preference to anxiety were not significant, whereas the path from ingroup affiliation preference to depression was significant for the EA, but not the MA, group.

## Discussion

Stronger ethnocultural identity often is associated with better psychological health. This relationship has been found among both ethnic minority and ethnic majority group members and across multiple ethnocultural groups within and outside of the US. In the current study, we proposed that social connection and emotion regulation might serve as mechanisms to explain such linkages. Although we cannot make definitive causal attributions, our data are largely consistent with the notion that, at least for women, psychological ties to one's ethnic and cultural group may influence mental health indicators through social connection and in turn emotion regulation processes.

Using path analysis, we found that among both MA and EA women, stronger ethnic identity and also greater comfort with mainstream culture were associated with stronger social connectedness, which in turn predicted expectancy for more effective negative emotion regulation, fewer self-reported depressive symptoms, and lower general feelings of anxiety. Preference for ingroup affiliation also was related to social connectedness in both groups, but in an unexpected direction: a stronger preference for same-ethnic affiliation was associated with a *weaker* sense of social connection, worse emotion regulation expectancy, more depressive symptoms, and higher anxiety. In addition to its direct effects on depression and anxiety, social connection also had indirect effects (through negative emotion regulation expectancy) on these outcomes. In other words, for both groups, social connectedness emerged as a centerpoint through which ethnocultural variables were associated with mental health indicators.

Direct paths from ethnocultural variables to negative emotion regulation expectancy were evident only in EAs (not MAs), and only for ingroup affiliation and mainstream comfort (not ethnic identity). In addition, negative emotion regulation expectancy was not a strong predictor of anxiety in this sample. Consequently, indirect paths from ethnocultural variables through negative emotion regulation to depression and anxiety were largely non-significant.

Finally, we found that ethnic identity and preference for ingroup affiliation were positively related among both MAs and EAs, but that this relationship was considerably stronger for MAs. Also among MAs, ethnic identity and ingroup affiliation each were inversely related to mainstream comfort, whereas this was not the case for EAs. These relationships among the ethnocultural variables have important implications for whether both groups are able to experience social connection and its potential mental health benefits to an equivalent extent. Below we discuss these findings and their limitations, implications, and relevance for intersections of clinical and cultural psychology.

### Benefits of ingroup and outgroup affiliation: the pivotal role of social connection

One goal of the present paper was to test more explicitly the role of social connection in the links between ethnocultural identity/affiliation and indicators of mental health. Much of the current literature implies that social connection plays a key role, as studies show that ethnocultural group identification and ingroup affiliation mitigate loneliness, provide social support, and/or offer a sense of community and belonging (Roberts et al., 1999; Galliher et al., 2011; Rivas-Drake, 2012). Nevertheless, framing this previous work explicitly in terms of social connection may be advantageous for understanding how ethnic identity benefits mental health. Also, the construct of social connection (or loneliness) is now well-known in social and affective neuroscience. We believe an integration of physiological perspectives into the minority mental health literature may advance the field by bringing to bear additional rich theoretical approaches. For example, along with conceptualizing ethnic identity in terms of identity formation (e.g., from an Eriksonian perspective; Phinney et al., 2007), new ideas about underlying mechanisms may be generated by considering ethnic identity and ingroup affiliation in terms of their possible evolutionary basis, such as the need for attachment and affiliation. Furthermore, our understanding of sociocultural processes has been enhanced by including brain- and body-based measures to complement self-report measures of subjective experience. Even when Latinos and Whites do not differ in family giving behavior, for example, Latinos show greater neural reward activation when giving to one's family vs. oneself (Telzer et al., 2010). Finally, our knowledge of the impact of loneliness and, conversely, social connection has been greatly enhanced by the inclusion of physiological measures and outcomes with psychosocial assessments (Hawkley and Cacioppo, 2010).

Considering social connection explicitly also is critical given extensive evidence that the psychological experience of loneliness (and, conversely, social connectedness) is highly predictive of emotional, emotion regulatory, and physical and mental health consequences (Schwartz and Olds, 1997; Hawkley and Cacioppo, 2010; Patterson and Veenstra, 2010; Luo et al., 2012). This psychological experience appears even more influential than the size of one's social network or available supports from that network. Models of our data underscore that social connection is a central mechanism through which ethnocultural identities—including with one's own group and the mainstream cultural group—relate to mental health, and in fact may be *part of* these identifications (i.e., *ethnocultural social connection*).

In both EA and MA participants, greater identification with one's culture of origin and also greater sense of belonging to mainstream culture were associated with greater social connectedness. Despite these same “ends,” there may be different meanings of the constructs involved, and different means through which these paths occur. For example, for MAs, greater ethnic identity is likely about a relationship with Mexican culture, whereas for EAs, greater ethnic identity may arise either with respect to a culture of origin more distant in time (e.g., a German background) or with respect to American culture (e.g., parents may have instilled a sense of ethnic pride as an “American”; Martinez and Dukes, 1997). Similarly, for MAs, greater comfort with mainstream culture may reflect stronger bicultural values and attitudes, whereas for EAs, it may simply reflect a more general sense of integration into society at large.

For MAs, the fact that both one's own group and the mainstream cultural group offer a sense of social connection is consistent with previous literature pointing to the benefits of bicultural identification (e.g., LaFromboise et al., 1993). However, given that ethnic identity and mainstream comfort were inversely related, many MAs may find it difficult to derive the benefits from both of these sources of social connection. In addition, it is possible that Latina college students with a stronger ethnic identity may be more likely to encounter and/or internalize stigma, discrimination, and a sense of marginalization (e.g., Castillo et al., 2006), therefore making it difficult to achieve the type of true bicultural identification that both the literature and our data suggest is beneficial for mental health.

Contrary to our hypotheses, for both MAs and EAs a stronger preference for affiliating with members of one's own group was associated with *less* of a sense of social connection. There are several possible explanations for this finding. First, *actual* interactions with ingroup members may be beneficial, whereas a *preference* for these interactions may not. For example, if a strong desire to socialize with members of one's own group is met with an unavailability of group members, it may create emotional emptiness or longing, particularly among MAs (Rivas-Drake, 2012). Second, preferential affiliation with members of one's own group may create fewer opportunities for establishing meaningful emotional connections with others by decreasing the number of people available in one's social environment. Third, we suspect our measure of ingroup affiliation preference, namely the *social affiliation* subscale of the SEE, also assesses negative intergroup attitudes. For example, items such as, “I find it easiest to trust people from my own ethnic group,” may imply a distrust of outgroup members. In this case, it may not have been the preference for ingroup members, but rather an uneasiness, about outgroup members that was associated with negative psychological outcomes. Support for this notion comes from the fact that positive intergroup attitudes among both majority and minority individuals are associated with psychological health (Phinney et al., 2007; Torres and Rollock, 2007).

In both of these ways—greater social connection from ethnocultural identity and mainstream comfort, and diminished social connection from preferences for ingroup members (possibly to the exclusion of outgroup members)—social connection may play a pivotal role in the relations between ethnocultural experience and mental health indicators.

### Ethnocultural social connection may enhance emotion regulation

Given its centrality to both interpersonal relationships and mental health, we expected emotion regulation to play a key role in the relationship between the social connection that comes from ethnocultural group identity/affiliations and mental health symptoms. We found that expectancies for effective emotion regulation were important insofar as they serve as a mechanism through which ethnocultural social connections exert their effects on mental health. There were direct relationships between two of our ethnocultural variables—mainstream comfort and ingroup affiliation—and negative emotion regulation expectancy for EAs, but for MAs, there were no direct relationships.

Empirical studies have not always found cultural differences in emotional responses, as might be expected based on anthropological and anecdotal evidence. Rather, values or attitudes about emotion (Tsai et al., 2006; Mauss et al., 2010), the fit between one's cultural context and emotions (Chentsova-Dutton et al., 2010; Perez and Soto, 2011) and, here, the influence of ethnocultural social connection, may offer more possibilities for culture to shape emotional processes. Further, ethnocultural social connection may be particularly important in bolstering the sense that it will be possible to pull oneself out of negative feelings when needed, which is what we measured in the current study. This positive expectancy—along with actual ability to manage emotions—is important for mental health (Benight and Bandura, 2004; Baumeister et al., 2007). It also may function as “antecedent-focused” emotion regulation (Gross, 1998), the beginning of the emotion regulatory cycle.

### Considering ethnocultural social connection in context

In this paper, we suggest that one's sense of ethnocultural identity and desire to interact with ingroup members, along with one's comfort in mainstream society, influence social connectedness and emotion regulation expectancies. We further suggest that these processes may be largely pan-cultural, similar to other fundamental processes such as attachment—and that they also hold the same potential to become dysfunctional. It may seem antithetical to the notion of cultural psychology to refer to a process as pan-cultural, as from this perspective, lumping cultural groups together is similar in its lack of specificity to saying we are studying humans. The construct of ethnic identity itself, however, has been conceptualized as largely pan-group, because it measures the psychological relationship to one's culture *irrespective of* specific cultural content (Phinney and Ong, 2007).

Nevertheless, ethnic identity undoubtedly can take on different meanings or implications depending on the particular group or context (also noted in Phinney and Ong, 2007). One distinction in this regard refers to minority vs. majority status. Ethnic minorities in the US share many aspects of experience as members of a non-dominant group, potentially facing discrimination or disempowerment, straddling multiple cultural worlds, and often working to establish a culture within a culture. There also are distinctions among the major ethnic minority groups in the US, as a result of the particular political histories or immigration statuses of these groups, and/or stereotypes held by outgroup members. For example, because Blacks/African Americans and Asian Americans are perceived differently and may hold different roles in US society, racial and ethnic identity may have different meanings and relationships to other psychological processes for these two groups (Helms, 2007; Phinney and Ong, 2007). Further, within these larger *ethnic* groups, such as *Hispanic/Latino*, there are multiple *cultural* groups, with more specific shared systems of meaning that have been transmitted intergenerationally (Matsumoto, 1993; Shweder et al., 2008).

In the present study, we included women of Mexican descent (MA) to test our model in an ethnocultural minority group. MA participants simultaneously held Mexican, Hispanic/Latino, and minority status (both gender and ethnic), each of which may have contributed in distinct ways to their experience (for a thorough review of Hispanic/Latino ethnic identity, with consideration of specific Latino subgroups, see Quintana and Scull, 2009). For example, Mexican culture typically is described as collectivistic and as emphasizing family values (familism), especially among women (Campos et al., 2008). Therefore, for Mexican and Mexican American women, a sense of social connection may be particularly beneficial, and its absence particularly problematic, because of the emphasis Mexican culture places on social connection. Again, though, in a study conducted by Gaines and colleagues (1997), *all* major ethnic minority groups reported stronger ethnic identity and also more strongly endorsed the cultural values of collectivism and familism than did White individuals, suggesting that ethnocultural social connection may have importance due to minority status—as opposed to, or in addition to, Mexican status.

Still, this is not to say that there are not nuances in the larger cultural and contextual forces that shape the relationships we found or in the meaning of these relationships. For Hispanics/Latinas in the US today, particularly in a border state such as Arizona, where data collection took place, there is a strong anti-immigrant political climate that may create heightened anxiety about racial/ethnic status. Despite the sizable MA population in the area where this study was conducted, being surrounded by members of the majority on a daily basis can have psychological consequences of its own (Perez and Soto, 2011). Such a sociopolitical climate may create unease about fully connecting with mainstream culture, particularly for Latinas with a strong ethnic identity (given the inverse relationship between mainstream culture and ethnic identity as noted above).

### Limitations

Our data are based on a cross-sectional survey of relatively healthy college women from two ethnic groups. This approach has several clear limitations with respect to sample size, generalizability, reliance on self-report, and causal inference. Here we discuss each of these in greater detail. First, although our full sample is a reasonable size, the group of MA participants should ideally be larger for testing our model. Therefore, although the path coefficients were similar in the MA subsample to those in the overall sample, these results should be replicated to ensure their reliability.

Second, we studied college students, who on average reported relatively low levels of depression and anxiety symptoms. Many studies on ethnic identity similarly have surveyed college students, which, on the one hand, we believe has limited the progress of this area of research. These findings are not automatically generalizable to a less well-educated population, or to a community or clinical population. On the other hand, the transition to college and to young adulthood in general (in Eriksonian terms, navigating the stages of “identity vs. role confusion” and “intimacy vs. isolation”) is a crucial time with respect to the process of ethnic identity, and the processes of social connection and emotion regulation as well. Understanding sub-clinical or relatively low levels of depression or anxiety may be useful with respect to understanding young adults' experience (e.g., stress management, academic achievement, risk for more severe clinical symptoms), although again, they are not a substitute for studying clinical populations.

In addition, we studied two ethnocultural groups that were based on self-identification as MA or EA. Although we assessed generational status, and we included only participants who attended high school in the US and were attending college at a major university in the US, we do not have additional information about ethnic and cultural background; therefore, our understanding of cultural influences on the processes of interest is necessarily limited (Roosa et al., 2008). Greater ethnic identity may reflect greater endorsement of cultural values; nevertheless, we do not know how much participants endorsed specific cultural values, and how these intersected with ethnic identity. Importantly, group-specific status (as MA vs. EA) was confounded with minority/majority status. For example, we cannot ascertain whether the relationships observed among our MA group were attributable to Mexican culture, minority status, or other factors. Further, some of our MA sample endorsed “White/European” as their race, whereas others endorsed “Other” and wrote in “Mexican, Hispanic,” or “Latino” as their race. This may reflect an artifact of the census categories, or it may reflect a meaningful difference based on ancestry, self-perceptions, or cultural influences (Waters, 1990; Navarro, 2012).

We also studied only women. As noted earlier, women have a higher prevalence of mood and anxiety disorders than men (Nolen-Hoeksema and Girgus, 1994; Lewinsohn et al., 1998; McLean et al., 2011) and are more likely than men to seek comfort from and to be emotionally affected by relationships (Gottman and Levenson, 1992; Taylor et al., 2000). Nevertheless, because ethnic identity may serve different functions for men and women (Jones and Galliher, 2007), it will be important to study the relationships discussed here in both men and women of different ethnocultural groups.

Another limitation is our reliance on self-report, including the assessment of preferences for ingroup affiliation, rather than actual affiliation, and for self-reported expectancies for emotion regulation, rather than actual emotion regulation (including its many facets). We argue that the subjective experience of ethnocultural group identities and preferences has a considerable psychological impact, and that similarly, perceptions of depression, anxiety, loneliness, and expected emotion regulation abilities matter with respect to shaping one's subjective experience and sense of distress. Nevertheless, self-reports of behavior are different from actual behavior.

Finally, because we used a cross-sectional sample to collect all of our data, we cannot make causal inferences. Path analysis allows us to determine only whether one set of modeled relationships is a better fit to the actual data than another set of such relationships. Therefore, it may be the case (for example) that individuals who felt more depressed or anxious had greater difficulty connecting emotionally with others, and in turn had greater difficulty establishing an ethnic identity, a sense of belonging to mainstream culture, and/or trusting outgroup members.

### Implications for intersections of clinical and cultural psychology

Over the past several decades, many scholars have noted a cultural shift in the US marked by “the waxing of the individual and the waning of the commons” [per Seligman's (1990) article by that name]. This shift has coincided with increases in narcissism, depression, anxiety, and loneliness (Seligman, 1990; Schwartz and Olds, 1997; Twenge and Foster, 2008), including among ethnocultural minority groups. Specifically, Hispanic and Asian college students, who are part of the two fastest-growing immigrant groups in the US, demonstrate trajectories of increasing narcissism similar to White students (Twenge and Foster, 2008). This is one of many examples of how adopting mainstream American cultural values, beliefs, attitudes, and practices may have negative consequences (Egolf et al., 1992; LaFromboise et al., 1993)—and yet these negative consequences can potentially be offset by simultaneously maintaining stronger ethnocultural ties (i.e., bicultural adaptation; LaFromboise et al., 1993; Berry, 1994). In addition, there may be a parallel process in which individuals in the US, in general, have become less trusting over time (Putnam, 1995; Schwartz and Olds, 1997). This diminished trust may also perpetuate a greater sense of social disconnect, especially if coupled with a reactionary over-reliance on one's own group, as our data suggest. Our findings, therefore, echo previous work indicating that an optimal level of ethnocultural connection, characterized by stronger ethnic identity as well as greater comfort with mainstream culture and a willingness to develop connections with outgroup members, is associated with indicators of mental health. This nevertheless is not always easy to achieve. Importantly, however, the current paper shows that stronger social connection is a clear bridge to these indicators. We use the term *ethnocultural social connection* to make explicit a process that, we believe, has been implied in the ethnic identity literature for many years.

Although ethnic identity has some roots in social identity theory (e.g., Tajfel, 1982), there arguably is a gap between the social psychological literature, which focuses primarily on implications of ingroup identity and affiliation for social, emotional, and intergroup processes, and the ethnic identity literature, which focuses on implications for psychological adjustment or mental health from the perspectives of counseling, educational, and developmental psychology. There may be an additional gap between this latter perspective and clinical psychology/psychopathology, as reflected in part by the rift between empirically-supported and culturally-sensitive therapies (Hall, 2001). However, both fail to fully address the issues that may facilitate or undermine successful treatment of ethnic minority clients (Kirmayer, 2001). In one sense, the ethnic identity literature itself may be viewed as a metaphorical ingroup that is central in its own domain, but marginalized in the larger context of mainstream psychology. The perspective offered here attempts to begin to bridge this gap.

Our findings reinforce the notion that addressing an absence of social connectedness may be a critical step toward enhancing mental health, and that fostering positive expectancies with respect to emotion regulation is yet another key aspect of this process. Making sense of and/or regulating one's emotions is a core aim of many current first-line empirical treatments, such as cognitive processing therapy for post-traumatic stress disorder, dialectical behavior therapy for borderline personality and other disorders, and even cognitive-behavioral therapy for depression. Although definitive causal inferences cannot be made with our data, we suggest that social connection may be an important avenue through which emotion regulation can be improved.

A cornerstone of psychotherapy efficacy is the therapy relationship, which itself may be healing largely because it provides social connection. Going forward, treatments may be wise to target social connection more specifically (Schwartz and Olds, 1997). One vehicle for this may be through sensitive consideration of clients' attitudes toward their culture of origin, mainstream culture, and ingroup/outgroup members. These issues may be particularly salient for ethnic minority students at universities and secondary schools, where much of the current ethnic identity literature is focused. Focusing on potential barriers to achieving a bicultural identity and to experiencing a sense of social connection in multiple aspects of one's life may be warranted. In addition, although a clear goal in the US is to improve minority mental health, our data suggest that developing multifaceted ethnocultural connections is important for majority (non-Hispanic White) individuals as well. This in turn may improve the ethnocultural experience for other groups by creating a more culturally aware majority group. Given that the National Institute of Mental Health is calling for a diagnostic shift from a focus on symptoms to a focus on processes (e.g., emotion, cognition, and social relationships), such an approach is appropriate and timely.

### Conflict of interest statement

The authors declare that the research was conducted in the absence of any commercial or financial relationships that could be construed as a potential conflict of interest.

## Footnotes

^1^The NMR scale was shortened in order to reduce burden on the participants. We also administered the Difficulties in Emotion Regulation Scale (DERS; Gratz and Roemer, 2004), and analyses using the DERS differed only slightly from the analyses we report here. We chose to report the current analyses rather than those using the DERS because of the much larger literature employing the NMR scale.

^2^We also added generational status, age, and household income as predictors, to determine whether their inclusion would substantively alter the models. Although generational status was related to the other ethnocultural variables, none of these variables added significant additional predictive power, and none altered the relations among the variables of interest. Therefore, we omitted them from further analysis.

^3^To examine whether there might be differences in the underlying construct of depressive symptoms between the MA and EA groups, we tested Model 3 in the MA sample using both the original CES-D subscales and the revised subscales for Latinos (Kim et al., 2011) in place of the full scale scores. No substantive differences were found in model fit between the alternate versions of the subscales.
